# Effect of dietary phosphorus content on milk production and phosphorus excretion in dairy cows

**DOI:** 10.1186/2049-1891-5-23

**Published:** 2014-04-22

**Authors:** Chong Wang, Zhen Liu, Diming Wang, Jianxin Liu, Hongyun Liu, Zhiguo Wu

**Affiliations:** 1 Institute of Dairy Science, Zhejiang University, Hangzhou, P. R. China; 2 College of Animal Science and Technology, Zhejiang Agriculture and Forestry University, Lin’an, Hangzhou, P. R. China; 3 School of Veterinary Medicine, University of Pennsylvania, 382 West Street Road, 19348 Kennett Square, USA

**Keywords:** Dairy cows, Milk production, Phosphorus excretion, Phosphorus requirement

## Abstract

**Background:**

Phosphorus (P) supplementation is costly and can result in excess P excretion. This study investigated the effects of reducing dietary P on milk production and P excretion in dairy cows over a full lactation.

**Method:**

Forty-five multiparous Holstein dairy cows were divided into 15 blocks according to expected calving date and previous milk yield, and assigned randomly to one of the three dietary treatments: 0.37, 0.47, and 0.57% P (DM basis); these P levels represent the NRC recommendations, Chinese recommendations, and the amount of dietary P commonly fed by Chinese dairy farmers, respectively. Average daily feed intake was calculated from monthly data on feed offered and refused. Milk yields of individual cows were recorded weekly, and milk samples were taken for analysis of protein, fat, solids-not-fat, lactose, and somatic cell count. Blood samples were collected on days −6, −3, 0, 3, 6 relative to calving, and then monthly throughout lactation, and analyzed for P and Ca concentrations. Spot samples of feces and urine were collected for 3 consecutive d during weeks 12, 24, and 36, and P concentrations were analyzed. Reproduction and health data were recorded.

**Results:**

Dietary P did not affect dry matter intake or milk yield (*P* > 0.10). Milk fat content was slightly higher in cows fed 0.37% P than in cows fed 0.47% P (*P* = 0.05). Serum concentrations of P and Ca did not reflect dietary P content (*P* > 0.10). Fecal and urinary P both declined linearly (*P* < 0.05) as dietary P decreased from 0.57 to 0.37%. Fecal P content was 25% less when dietary P was 0.37% compared to 0.57%. Health events and reproductive performance were not associated with dietary P content (*P* > 0.05).

**Conclusions:**

Lowering dietary P from 0.57 to 0.37% did not negatively affect milk production, but did significantly reduce P excretion into environment.

## Background

Phosphorus (**P**) is an essential nutrient for animal production and reproduction, and dairy producers often feed inorganic supplements to increase the P content of the diet. However, P supplementation is costly and can result in excess P excretion, contributing to the eutrophication of waterways. A study conducted by Wu et al. [1] showed that dietary P supplementation is not necessary for most dairy herds in the US. The latest NRC [2] recommendation reduced the P requirements for lactating cows from previously recommended values [3]. These requirements range from 0.32% (DM basis) for cows milking 25 kg/d to 0.38% for cows milking 54 kg/d.

Cows in China produce approximately 4,000 kg/yr of milk, compared to the 9,000 kg/yr produced by dairy cattle in the US [4]. In East China, milk production is higher, averaging 7,200 kg/yr [5]. The P requirement recommended by the Chinese feeding standard is 0.45% for this level of production, higher than the 0.37% currently recommended by the NRC [2]. A recent survey of Chinese dairy farms [4] found that as much as 0.55% P was fed on most of the farms contacted. These findings suggest that Chinese dairy cows are currently fed too much P. Therefore, the purpose of this study was to investigate whether reducing dietary P over a full lactation affects the lactation performance of dairy cows in China.

## Materials and methods

### Animals, diets and experimental design

The use of animals was approved by the Animal Care Committee of Zhejiang University, Hangzhou, China. The experiment was conducted on a farm with a 1,000-cow herd fed TMR. Forty-five multiparous Holstein dairy cows (609.0 ± 23.0 kg body weight) were selected and divided into 15 blocks of three cows each according to expected calving date and milk yield during the previous lactation (6,381.0 ± 145.0 kg), and then assigned randomly within block to one of the following dietary treatments: 0.37 (**LP**), 0.47 (**MP**), and 0.57% P (**HP**) (Table 1). These P levels were obtained by altering the calcium phosphate and dicalcium phosphate content of the diets (Table 1). The amount of P in the LP diet was close to the level recommended by the NRC, the MP diet contained P at a level similar to the Chinese feeding standard recommendations [6], and the HP diet represented the amount of P commonly fed by milk producers in China [4]. All diets contained 45% forage, primarily corn silage and grass hay. The cows were fed their assigned diets for the duration of their lactation. Diet formulations (as-fed basis) were adjusted weekly to compensate for changes in the DM content of the ingredients.

**Table 1:** Ingredients and chemical composition of diets (DM basis)

**Items**	**Dietary treatment**^**1**^		
	**LP**	**MP**	**HP**
Ingredient,%			
Ground corn grain	22.0	21.9	21.8
Wheat bran	4.1	4.1	4.1
Soybean meal	4.2	4.2	4.2
Sesame meal	3.2	3.1	3.1
Cotton meal	3.0	2.9	2.9
Cotton seed	2.1	2.1	2.1
Calcium carbonate	0.9	0.6	0.2
Salt	0.4	0.4	0.4
Dicalcium phosphate	0.0	0.5	1.1
Corn silage	20.1	20.7	20.7
Grass hay	16.9	16.9	16.9
Alfalfa hay	7.6	7.6	7.7
DDGS	8.3	8.3	8.3
Apple pulp	6.0	6.0	6.0
Premix^2^	0.5	0.5	0.5
Chemical composition^3^			
Dry matter,%	57.9	57.9	57.9
P,%	0.37	0.47	0.57
Ca,%	0.74	0.74	0.74
CP,%	15.2	15.2	15.2
NDF,%	32.6	32.8	32.9
NEL, Mcal/kg	1.51	1.51	1.51

^1^LP = low P; MP = medium P; and HP = high P.

^2^Formulated to provide (per kg of DM) 1,000,000 IU of vitamin A, 200,000 IU of vitamin D, 1,250 IU of vitamin E, 14,000 mg of Zn, 100 mg of Se, 180 mg of I, 3,000 mg of Fe, 40 mg of Co, 3,000 mg of Mn, and 3,000 mg of Cu.

^3^All values were the actually determined averages from the monthly TMR analyses with the exception of the NEL, which was estimated using tabular values in the Chinese standard [6].

The experiment was conducted from January 2009 until February 2010. Cows were housed in a tie-stall barn with free access to water, offered TMR three times daily ad libitum (5 to 10% refusal), and milked at 0630 h, 1430 h, and 1930 h. Average daily feed intake was calculated from monthly data on feed offered to and refused by individual cows. After completion of a 40 wk lactation cows were dried off or removed from the experiment. As a normal herd management practice, some cows were dried off after less than 40 wk of lactation in order to insure an 8-wk dry period before the next lactation, or were removed from the experiment when their milk yield dropped to 9 kg/d or when they developed significant health problems. During the dry period following the trial, all cows were fed a standard dry cow diet with a P level of 0.40%.

### Sampling, measurement, and analyses

Feed ingredients and TMR were sampled monthly (every 4 wk). Samples were dried at 60°C for 48 h, ground and passed through a Wiley mill with 1-mm screen, and analyzed for neutral detergent fiber (NDF) [7], DM, calcium (**Ca**), crude protein (CP) [8], and P [9]. A certified commercial P solution (VHG Labs, Inc., Manchester, NH) was used as a calibration standard. Accuracy of the analysis was assured by using additional commercial standards (Standard Reference Material 1570a-spinach leaves, and 8436-durum wheat flour; National Institute of Standards and Technology, Gaithersburg, MD) and by interlaboratory comparison of reference samples, which revealed differences of <5%. Chemical analyses were based on DM measurements made at 105°C.

Milk yields of individual cows were recorded weekly. At the same time, daily milk samples were taken in amounts proportional to yield (4: 3: 3, composite) and analyzed for protein, fat, solids-not-fat (SNF), lactose and somatic cell count (SCC).

Blood samples (10 mL) were collected 3 h after feeding on days −6, −3, 0, 3, 6 relative to calving, and then monthly throughout lactation. Blood samples were centrifuged at 2,200 × g for 15 min, and serum P and Ca concentrations were analyzed by Nanjing Jiancheng Bioengineering Institute (Nanjing, China) according to AOAC procedures [8].

Spot samples of feces and urine were collected three times during the experiment (on 3 consecutive d during weeks 12, 24, and 36) as described by Wu et al. [1]. Both fecal and urinary P levels were analyzed using the same method as for feed analysis.

Reproduction and health data were recorded. Visual detection of estrus was performed by the farm technician while the cows were in the holding area before milking, and was based on standing, mounting, and mucus discharge. Cows were inseminated at their first estrus after 52 d postpartum and at every estrus thereafter until conception occurred. Pregnancy was confirmed by rectal palpation. Conception rate at the first AI was the percentage of cows that conceived on first AI. Pregnancy rate was defined as the number of cows confirmed pregnant divided by the number of cows bred. Cows were identified as nonbreeders when they failed to become pregnant by 230 d in milk. These cows were not used in the calculation of pregnancy rates.

### Statistical analysis

Measured milk yield, milk component percentages, and DMI were reduced to monthly averages. These averages, and the blood Ca and P concentrations, between-diet differences were assessed by repeated measures analysis using Proc MIXED in SAS (SAS institute Inc., Cary, NC); treatment, time, treatment × time, and block were included as effects in the model. An AR (1) covariance structure was utilized. Milk production during the previous lactation and serum Ca and P concentrations at −6 d relative to calving were included as covariates in the models for analysis of milk yield and serum variables, respectively. Other data were analyzed using the general linear models procedure in SAS. Treatments were also tested for linear and quadratic effects by orthogonal polynomial contrasts. Categorical data were analyzed for treatment effects using Proc FREQ (SAS) with a chi-square and Fisher’s exact test.

Probability values of *P* < 0.05 were used to define statistically significant results, with statistical trends being defined at *P* < 0.10. Statistics related to block are excluded from discussion because of lack of significance of the term.

## Results and discussion

### Diet composition and feed intake

Treatment diets were formulated to differ only in P content, which was 0.37, 0.47, or 0.57% (DM basis, Table 1). The other ingredients of the diets were identical in their nutrient content throughout the experiment; therefore, all diets had the same CP, NDF, and Ca (*P* > 0.05).

**Figure 1:**
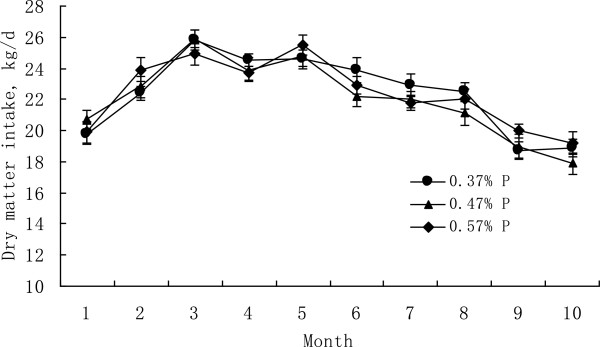
**Change in dry matter intake of cows fed diets containing 0.37, 0.47, or 0.57% of P over a full lactation period.** Means for the respective treatments over the entire lactation were 22.4, 22.0, and 22.4 kg/d (P = 0.12), with no treatment by sampling period interaction (P= 0.43). Values are expressed as mean ± SE.

Feed intake did not differ among treatment groups (Table 2, Figure 1). Valk and Sebek [10] reported that feeding cows a 0.24% P diet reduced DMI during the dry period following the first year of the study, and during the second lactation. However, most published studies suggest that dietary P content does not affect DMI [10,11] unless the amount is so low (<0.25%) that the activity of rumen microbes is impaired [12]. Clearly, our LP diet (0.37% P) was not so low in P that it reduced DMI.

**Table 2:** Milk production of cows fed diets differing in P content

**Items**^**1**^	**Dietary treatments**^**2**^			**SEM**	***P***^**3**^				
	**LP**	**MP**	**HP**		**T**	**t**	T*t	**L**	**Q**
DMI, kg/d	22.4	22.0	22.4	0.77	0.12	0.03	0.43	0.69	0.65
Milk yield, kg/d	21.5	20.7	22.0	1.03	0.63	0.01	0.03	0.66	0.61
3.5% FCM, kg/d	20.8	19.7	21.5	0.62	0.09	0.01	0.47	0.54	0.60
Milk protein,%	3.33	3.30	3.28	0.05	0.77	0.01	0.02	0.79	0.68
Milk fat,%	3.71	3.41	3.61	0.09	0.05	0.01	0.11	0.36	0.15
Milk lactose,%	4.61	4.69	4.66	0.03	0.46	0.03	0.29	0.55	0.71
Milk SNF,%	8.77	8.74	8.72	0.06	0.25	0.03	0.33	0.59	0.55
SCC, 10^3^/mL	302	350	231	44.9	0.05	0.58	0.29	0.35	0.03

^1^DMI = Dry matter intake; FCM = Fat corrected milk; SNF = Solid not fat; SCC = Somatic cell count.

^2^LP = low P; MP = medium P; and HP = high P.

^3^T, effect of treatment; t, effect of time; T*t, treatment by time interaction; L, linear effect; Q, quadratic effect.

### Lactation performance

Eight cows (2, 3, and 3 in the LP, MP, and HP groups, respectively) were culled prior to completing lactation. To obtain 280-day lactation information for these cows, the milk yields from the last five weekly averages were used to extrapolate estimates for the missing weeks by linear regression.

**Figure 2:**
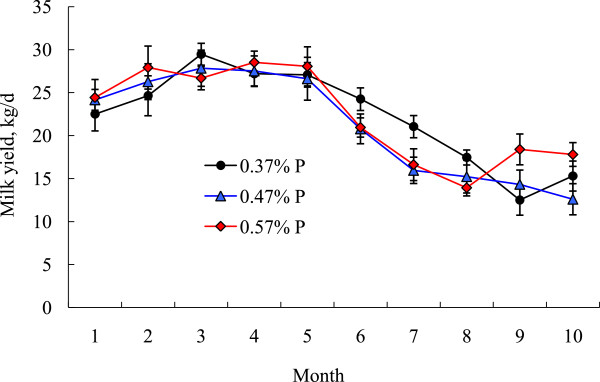
**Change in milk yield of cows fed diets containing 0.37, 0.47, or 0.57% of P over a full lactation period.** Means for the respective treatments over the entire lactation were 21.5, 20.7, and 22.0 kg/d (P=0.63), respectively, with a treatment by sampling period interaction (P = 0.03). Values are expressed as mean ± SE.

The lactation curves in Figure 2 show that during the first 5 mo milk yield was similar (*P* > 0.10) among treatment groups. During months 6, 7, and 8, the average milk yield was higher in the LP group than in the other 2 groups (20.9 vs. 17.3 and 17.2 kg/d, P < 0.05). This relationship was reversed during the following 2 mo (treatment by time interaction, *P* <0.05). The lower milk yield of the cows fed the two higher P amounts during months 6 to 8 was unexpected, but similar observations were reported by Carstairs et al. [13], who showed that cows receiving 35% more P than required produced less milk than those fed according to the NRC recommendation [3]. Average daily milk yield did not significantly differ among treatment groups (21.5, 20.7, and 22.0 kg for the LP, MP, and HP groups, respectively; *P* > 0.05). Similarly, Wu and Satter [14] reported no difference in milk production over a complete lactation when cows were fed low (0.31 to 0.38%) or high (0.44 to 0.48%) dietary P. Lopez et al. [15] also reported that milk production was the same for cows fed 0.33 or 0.46% P.

Milk protein content was 3.33, 3.30, and 3.28% for the LP, MP, and HP groups, respectively (Table 2), and there were no significant differences among treatments (*P* > 0.1). Milk fat was lowest in the MP group (*P* = 0.05); overall, however, no linear or quadratic effect was observed. Similarly, there were no significant differences in milk lactose, fat-corrected milk (FCM), SNF, or SCC (*P* > 0.05). These observations are consistent with previous reports in the literature [16], which indicate that varying dietary P from 0.37 to 0.57% does not affect milk composition.

### Serum Ca and P

Mean serum Ca (2.05 to 2.06 mmol/L) and P (1.21 to 1.28 mmol/L) concentrations were similar among treatment groups before calving, when all animals were receiving the same diet. Serum Ca and P remained similar (2.00 to 2.04 mmol/L for Ca and 1.20 to 1.25 mmol/L for P) after calving for the entire lactation period, even though the diets differed in P content (Table 3, *P* > 0.1). Peterson et al. [17] reported that serum P concentrations ranged from 2.09 to 2.17 mmol/L in dairy cows fed diets containing 0.21 to 0.44% P. Knowlton et al. [18] found that serum P concentrations increased from 1.26 to 1.83 mmol/L when dietary P content increased from 0.34 to 0.67%. Dietary Ca concentration did not affect P balance in a 20 wk experiment in which the serum P concentrations ranged from 1.65 to 1.74 mmol/L [19]. The serum concentrations of P that we found in this study were consistent with results reported by Liu [4], who found serum P concentrations of 1.23 to 1.29 mmol/L in cows in East China. Although serum P concentrations do decline if cows receive inadequate P in their diet [1,18], Valk and Sebek [10] suggested that 0.28% dietary P is sufficient for cows producing 9,000 kg/yr of milk. This is substantially less than 0.37%, the lowest amount used in the present study, and our data suggest that the cows receiving 0.37% dietary P were not deficient in P. This observation is supported by the serum Ca data, which showed no difference among treatment groups. In a study in sheep, Breves et al. [20] found that serum Ca increases during P deficiency.

**Table 3:** Serum Ca and P of cows fed diets differing in P content

**Items**	**Dietary treatments**^**1**^			**SEM**	***P***^**2**^				
	**LP**	**MP**	**HP**		**Trt**	**t**	T*t	**L**	**Q**
Serum Ca, mmol/L									
Prepartum	2.05	2.06	2.06	0.27	0.92	0.28	0.78	0.56	0.57
Postpartum	2.03	2.04	2.00	0.13	0.87	0.32	0.66	0.55	0.61
Serum P, mmol/L									
Prepartum	1.21	1.22	1.28	0.04	0.34	0.12	0.84	0.49	0.60
Postpartum	1.25	1.23	1.20	0.07	0.60	0.22	0.74	0.43	0.58

^1^LP = low P; MP = medium P; and HP = high P.

^2^T, effect of treatment; t, effect of time; T*t, treatment by time interaction; L, linear effect; Q, quadratic effect.

### Fecal and urinary P excretion

**Figure 3:**
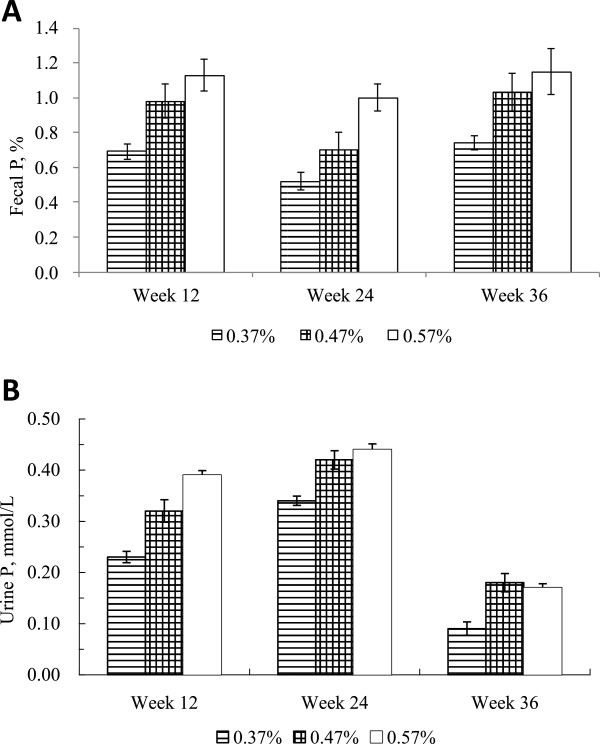
**Concentration of fecal P (A) and urinary P (B) in lactation dairy cows fed diets containing 0.37, 0.47, or 0.57% of P measured during different stages of lactation.** Values are expressed as mean ± SE.

The excretion of P during peak, middle, and late lactation reflected dietary P concentrations (*P* < 0.05, Table 4, Figure 3). Fecal and urinary P both declined linearly as dietary P decreased from 0.57 to 0.37%. These results suggest that reducing the amount of P fed to dairy cows in China from current levels to an amount consistent with NRC recommendations would reduce P excretion by 40% without affecting milk production. Tallam et al. [21] found that fecal P content was 29% lower in cows fed 0.35% P than in cows fed 0.47% P. Similar reductions in fecal P were found when dietary P was reduced [22,23]; such a reduction would have a significant impact on the environment.

**Table 4:** Fecal and urinary P concentrations at different stages of lactation in cows fed diets differing in P content

**Item**	**Dietary treatment**^**1**^			**SEM**	***P***^**2**^		
	**LP**	**MP**	**HP**		**T**	**L**	**Q**
Fecal P,% of DM							
Peak lactation	0.69	0.98	1.13	0.031	0.01	0.02	0.45
Middle lactation	0.52	0.70	1.00	0.102	0.03	0.04	0.39
Late lactation	0.74	1.03	1.15	0.085	0.02	0.02	0.26
Urine P, mmol/L							
Peak	0.23	0.32	0.39	0.011	0.01	0.01	0.44
Middle	0.34	0.42	0.44	0.018	0.01	0.03	0.29
Late	0.36	0.44	0.50	0.008	0.01	0.04	0.30

^1^LP = low P; MP = medium P; and HP = high P.

^2^T, effect of treatment; L, linear effect; Q, quadratic effect.

### Health problems and reproductive performance

There was no association between health events and dietary P content (Table 5). Reproductive performance also did not differ among treatment groups (*P* > 0.05; Table 5). Although, the present study did not use a sufficient number of animals to draw definite conclusions about dietary P content and animal health or reproductive performance, these data may be useful when combined with results from similar experiments. Several other studies have also showed no effect of dietary P on reproduction [14,21,24]. Noller et al. [25] reported similar reproductive performance in cows fed 0.22 or 0.32% P. Call et al. [11] reported that feeding diets containing 0.24, 0.32, or 0.42% P in their 12-month trial did not affect reproductive performance. In many parts of the world, dairy producers feed high dietary P to cows with the belief that P can improve reproductive performance [4]; however, our results indicate that reducing dietary P to the amounts recommended by the NRC [2] or the Chinese feeding standard [6] would not affect animal health or reproductive performance.

**Table 5:** Health events and reproductive performance of cows fed diets differing in P content

**Item**	**Dietary treatments**^**1**^			***P***
	**LP**	**MP**	**HP**	
Mastitis	3	2	2	0.63
Foot rot	3	4	3	0.72
Retained placenta	4	3	3	0.57
Displaced abomasum	3	2	1	0.64
Udder edema	2	3	3	0.56
Ketosis	3	2	2	0.63
Dystocia	1	0	1	0.55
Total health problems	19	16	15	0.43
Proportion of cows that conceived,%	80.2	88.5	70.5	0.54
Proportion of cows pregnant,%	75.9	77.5	72.0	0.66
Weight of calves, kg	35.4	37.1	35.8	0.64

^1^LP = low P; MP = medium P; and HP = high P.

## Conclusions

Lowering the dietary P content from 0.57 to 0.37% (NRC recommendation) did not negatively affect milk production in Chinese dairy cows, but did significantly reduce P excretion. The results of this study suggest that a dietary P content of 0.37% is sufficient for cows producing 21.5 kg/d of milk during lactation. Depending on the feed ingredients used, this concentration of P can be obtained without the addition of inorganic P supplements to the feed.

## Competing interests

The authors declare that they have no competing interests.

## Authors’ contributions

JXL and ZG Wu conceived the study and designed the experiment. CW, ZL, and HYL carried out the statistical analysis and drafted the manuscript. CW, ZL, and DMW performed the animal experiments. All authors read and approved the final manuscript.
